# Resuscitative thoracotomy in blunt traumatic cardiac arrest

**DOI:** 10.1186/s13049-022-01010-8

**Published:** 2022-04-25

**Authors:** Benjamin Stretch, Denise Gomez

**Affiliations:** 1 Queen Mary University London, London, UK; 2 Barts And The London school of Anaesthesia, London, UK

Many thanks to EHAAT for publishing their case series showing consistent delivery of resuscitative thoracotomy (RT) in a wide range of clinical scenarios [1]. Although sadly none of the patients survived, our understanding of traumatic cardiac arrest has been improved by the study. The majority (26/44) of RTs were performed in blunt trauma—a less well recognised indication for RT, with a small number of single case reports of survivors and multiple case series from around the world reporting dismal outcomes [2]. As a result, if there is a survival benefit of RT in blunt traumatic cardiac arrest, the NNT may be more than the 26 RT’s performed. The indications for blunt thoracotomy are poorly characterised as shown by a study from Nevins and colleagues, which showed great variation in standard operating procedures across UK pre-hospital services [2].

European Resuscitation Council (ERC) guidelines [3] recommend RT for relieving tamponade and aortic control in subdiaphragmatic haemorrhage in the context of appropriate Expertise, Equipment, Environment and Elapsed time (Fig. 1). In actively deteriorating trauma patients, particularly in the rural setting, there are limited treatment options for active non-compressible haemorrhage. An important finding from this study is that 15% of patients in blunt traumatic cardiac arrest had evidence of cardiac tamponade on RT, which may represent a reversible cause in some cases – however none of these patients survived and will have suffered more complex injury patterns than isolated tamponade.

**Fig. 1:**
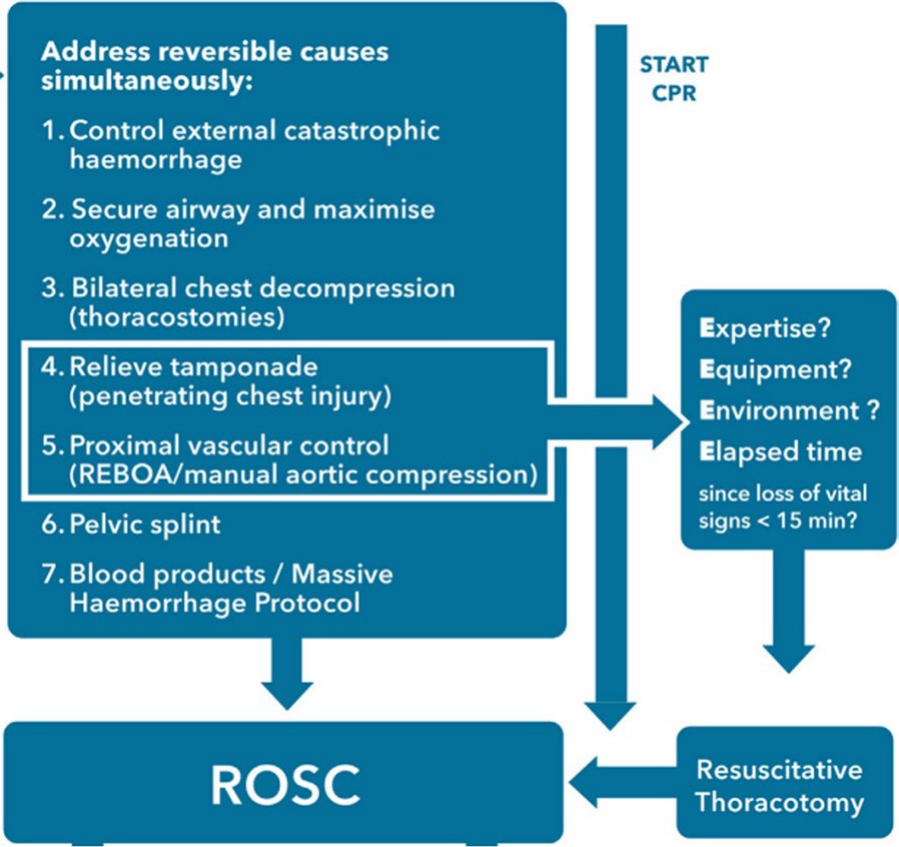
European Resuscitation Council guidelines on Traumatic Cardiac Arrest

The Royal College of Emergency Medicine (RCEM) are more pessimistic, stating that immediate surgical support and an onwards chain of survival are required following RT—otherwise the procedure is likely to be futile [4]. A challenge from this case series is geographical location of the incidents, with long transfer times resulting in only 6 of the 44 patients being stable enough for primary transfer to the major trauma centre. The “Trauma Emergency Thoractomy for Resuscitation In Shock” (TETRIS) study is an ongoing national audit on UK RT practice and may help identify which patients (if any) may benefit. Positive prognostic factors are likely to include on-scene expertise at the time of cardiac arrest with immediate RT; cardiac tamponade rather than exsanguinating haemorrhage; concurrent damage control resuscitation including balanced transfusion and temperature management; short transfer time to the Major Trauma Centre with early targeted surgical intervention; otherwise survivable injuries and absence of traumatic brain injury.

## Data Availability

Not applicable.

## Abbreviations

EHAAT: Essex and herts air ambulance trust (Title)
RT: Resuscitative thoracotomy
ERC: European resuscitation council
RCEM: The royal college of emergency medicine
TETRIS: Trauma emergency thoractomy for resuscitation in shock
