# Supplementary material for: Resuscitative thoracotomy in blunt traumatic cardiac arrest
Source: Scand J Trauma Resusc Emerg Med. 2022 Apr 25;30:30. doi: 10.1186/s13049-022-01010-8 (PMC9036700; doi:10.1186/s13049-022-01010-8)
Supplement: Related texts

# Reply: Authors’ Response

**Authors:** Phillip Almond, Sarah Morton, Matthew OMeara, Neal Durge

**Affiliations:** Essex and Herts Air Ambulance, Earls Colne, Colchester, CO6 2NS Essex UK

We are grateful to Stretch and Gomez for their feedback. We agree that the timeline for RT remains a challenge, particularly in our environment. We further agree about the current state of the literature landscape and hence why we wanted to share our findings. The point relating to Number Needed to Treat (NNT) is well made and we would go further in agreement by stating a survival rate of 1.4% would yield an NNT of 71—approximately three times the number in our blunt RT series [5]. In short, you have to kiss a lot of frogs!

Other systems have demonstrated that survival is possible, even in the face of long transfer times where blunt force trauma has resulted in cardiac tamponade [6]. It is perhaps a quirk of the rarity of such procedures which brings difficulty in studying or publishing such success. We wholeheartedly commend, support and contribute to the prospective trainee-led TETRIS STUDY, which is now recruiting.
